# Diseases of the Skin

**Published:** 1894-08

**Authors:** 


Diseases of the Skin. An Outline of the Principles and Practice of Dermatology. By Malcolm Morris, Surgeon to the Skin Department, St. Mary's Hospital, London, etc., etc. With eight chromo-lithographs and seventeen wood-cuts. Philadelphia: Lea Bros. & Co. 1894.

A handy little book, pleasingly written and well printed, designed to give briefly the clinical facts and accepted etiology of diseases of the skin. In the third chapter will be found a useful review of the general methods of diagnosis, and the uses to be made of presumptive evidence, thus enabling the student to reach a working diagnosis. Treatment and pathological details are made secondary to diagnosis, and the reader is referred to larger works.

